# DNA recovery from archived RDTs for genetic characterization of *Plasmodium falciparum* in a routine setting in Lambaréné, Gabon

**DOI:** 10.1186/s12936-019-2972-y

**Published:** 2019-10-02

**Authors:** The Trong Nguyen, Brice Nzigou Mombo, Albert Lalremruata, Erik Koehne, Rella Zoleko Manego, Lia Betty Dimessa Mbadinga, Ayola Akim Adegnika, Selidji Todagbe Agnandji, Bertrand Lell, Peter Gottfried Kremsner, Thirumalaisamy P Velavan, Michael Ramharter, Benjamin Mordmüller, Ghyslain Mombo-Ngoma

**Affiliations:** 1 Institute of Tropical Medicine, University of Tübingen, Wilhelmstraße 27, 72074 Tübingen, Germany; 2 German Center for Infection Research, University of Tübingen, Wilhelmstraße 27, 72074 Tübingen, Germany; 3 Centre de Recherches Médicales de Lambaréné and African Partner Institution, German-Center for Infection Research, Lambaréné, Gabon; 4 Vietnamese-German Center for Medical Research, Hanoi, Vietnam; 5 Department of Tropical Medicine, Bernhard Nocht Institute for Tropical Medicine & I. Department of Medicine University Medical Center Hamburg-Eppendorf, Hamburg, Germany; 6 Division of Infectious Diseases and Tropical Medicine, Department of Medicine I, Medical University Vienna, Vienna, Austria; 7 Fondation Congolaise pour la Recherche Médicale, Brazzaville, Congo

**Keywords:** Malaria, *Plasmodium falciparum*, RDT, *msp1*, *pfcrt*, Gabon, Capillary electrophoresis

## Abstract

**Background:**

Rapid diagnostic tests (RDTs) have been described as a source of genetic material to analyse malaria parasites in proof-of-concept studies. The increasing use of RDTs (e.g., in focal or mass screening and treatment campaigns) makes this approach particularly attractive for large-scale investigations of parasite populations. In this study, the complexity of *Plasmodium falciparum* infections, parasite load and chloroquine resistance transporter gene mutations were investigated in DNA samples extracted from positive RDTs, obtained in a routine setting and archived at ambient temperature.

**Methods:**

A total of 669 archived RDTs collected from malaria cases in urban, semi-urban and rural areas of central Gabon were used for *P. falciparum* DNA extraction. Performance of RDTs as a source of DNA for PCR was determined using: (i) amplification of a single copy merozoite surface protein 1 (*msp1*) gene followed by highly sensitive and automated capillary electrophoresis; (ii) genotyping of the *pfcrt* gene locus 72–76 using haplotype-specific-probe-based real-time PCR to characterize chloroquine resistance; and, (iii) real-time PCR targeting 18S genes to detect and quantify *Plasmodium* parasites.

**Results:**

Out of the 669 archived RDTs, amplification of *P. falciparum* nucleic materials had a success rate of 97% for 18S real-time PCR, and 88% for the *msp1* gene. The multiplicity of infections (MOI) of the whole population was 2.6 (95% CI 2.5–2.8). The highest number of alleles detected in one infection was 11. The MOI decreased with increasing age (β = − 0.0046, p = 0.02) and residence in Lambaréné was associated with smaller MOIs (p < 0.001). The overall prevalence of mutations associated with chloroquine resistance was 78.5% and was not associated with age. In Lambaréné, prevalence of chloroquine resistance was lower compared to rural Moyen-Ogooué (β = − 0.809, p-value = 0.011).

**Conclusion:**

RDT is a reliable source of DNA for *P. falciparum* detection and genotyping assays. Furthermore, the increasing use of RDTs allows them to be an alternative source of DNA for large-scale genetic epidemiological studies. Parasite populations in the study area are highly diverse and prevalence of chloroquine-resistant *P. falciparum* remains high, especially in rural areas.

## Background

Despite being treatable, malaria continues to be one of the major health problems in sub-Saharan Africa, with an estimated 219 million cases and 435,000 deaths in 2017 [1]. The clinical course can vary significantly between individuals, with many determinants remaining to be identified. Effective control and elimination require integrated multilayer strategies, including prompt diagnosis, appropriate chemotherapy, and case management [2].

Reliable, appropriate and timely diagnosis of febrile malaria patients is crucial, especially those caused by *Plasmodium falciparum*. This is achieved by microscopy of thick blood smears and/or using a malaria rapid diagnostic test (RDT) [2]. In addition, nucleic acid testing (NAT) is used to detect low-density *Plasmodium* infections in epidemiological studies aimed at surveillance of malaria control and elimination. The DNA template for NAT is usually recovered from anti-coagulant (EDTA)-treated blood or blood spotted on filter paper [3] and sometimes from thick blood smears [4]. Venous fresh blood sampling requires special storage conditions and training in venipuncture. Additionally, the use of venous blood samples leads to somewhat lower diagnostic sensitivity compared to capillary blood [5]. Capillary sampling is usually used for thick blood smears and sampling for RDTs or filter paper. Quality of DNA recovered from thick blood smears is low compared to filter paper and fresh blood [6] and the use of filter paper has to be prospectively planned, since it is not part of routine diagnostics. RDTs support rational and timely use of anti-malarial drugs in field settings, particularly if reliable microscopy is not available [7, 8]. The ease of use, low cost, and performance of RDTs has led to an increase in sales of these supplies in Africa from 240 million in 2015 to 269 million in 2016 [8]. Furthermore, successful amplification of DNA recovered from RDTs was observed [9–12] and the PCR detection rate in DNA extracted from RDTs is similar to that from filter paper [13]. Consequently, RDTs potentially represent an ideal source for large-scale retrospective analyses of parasite populations.

Parasite genotyping is essential for discrimination of new clones, from existing ones, to predict treatment outcomes (recrudescence *vs* re-infection). Additionally, it may provide evidence to understand the transmission dynamics in areas where there is perennial transmission of malaria. Natural infections can consist of multiple genetically distinct parasite strains co-infecting a single host. Such co-infections are reported as ‘multiplicity of infection’ (MOI). MOI is an indicator of transmission intensity [14] and may be used for molecular monitoring of any large-scale interventions. Genotyping approaches using conventional PCR targeting length polymorphic markers has been recommended to be used for assessment of efficacy of novel drugs [15]. The performance of these approaches varies between laboratories and mainly relies on the resolution of sizing systems. Despite the limitations: low resolution and time consuming, agarose gel electrophoresis is the most popular method for sizing due to its relatively low cost. A previous study showed capillary electrophoresis has better resolution and accurate estimation of genotypes than gel electrophoresis; this increased precision is critical especially in new anti-malarial drug trials to assess the efficacy of the intervention [16].

In the Republic of Gabon, artemisinin-based combination therapy (ACT) replaced chloroquine (CQ) as the first-line drug for the treatment of uncomplicated *P. falciparum* malaria in 2005. Since then, two studies have reported on the circulating genotypes based on merozoite surface protein 1 (MSP1) genotyping from Libreville in 2011–2012 [17] and from Oyem-Owendo for years 2008–2009 [18]. For Lambaréné, where there is perennial transmission, data on parasite genotypes exist for years 1995–1996 [19, 20] and CQ resistance for years 2001–2002 [21, 22], before the introduction of ACT. A few studies recently documented a differential distribution of CQ-resistant alleles among different geographical regions within Gabon [23, 24]. Yet, another study concluded that despite the implementation of ACT and the withdrawal of CQ, no significant difference in the prevalence of CQ-susceptible wild-type haplotype CVMNK was observed in parasite isolates collected between 2011 and 2014 [25]. Studies at regular intervals are required for molecular surveillance for anti-malarial drug resistance, including CQ [26].

Against this background, this study utilized a large cohort of individuals inhabiting Lambaréné, Fougamou and the respective adjacent rural areas, and aimed at investigating the performance of archived RDTs as a source of DNA for different molecular tests. Additionally, the study aimed to characterize parasite diversity, measured by MOI, and to determine the distribution of genetic polymorphisms in the *P. falciparum* CQ resistance transporter (*pfcrt*) gene locus.

## Methods

### Study site and samples

The study was conducted at the *Centre de Recherches Médicales de Lambaréné* (CERMEL), Lambaréné, Moyen-Ogooué, Gabon in July 2018. The study region is within the equatorial rainforest and highly endemic for falciparum malaria, which is perennial with little seasonal variation [27]. Local strains of *P. falciparum* show high levels of resistance against CQ and sulfadoxine-pyrimethamine [22, 28].

RDTs were collected from June 2017 to July 2018 during the screening process of an ongoing clinical trial (NCT03201770) and routine activities. The inclusion and exclusion criteria can be retrieved from the clinical trial registry NCT03201770. Briefly, participants of all ages and both genders were included if they presented with uncomplicated malaria, weight > 5 kg, and provided a signed informed consent. They were excluded in case of hepatic injury, known allergy to study drugs, and pregnant or lactating women.

Participants were tested for malaria using three different RDTs (VIKIA^®^ Malaria Ag Pf/Pan, IMAccess, Lyon, France; Paracheck Pf^®^, Orchid Biomedical Systems, Goa, India; SD BIOLINE Malaria Ag P.f/Pan Standard Diagnostics Inc, Hagal-Dong, Korea). All RDTs were WHO prequalified to detect *P. falciparum* antigens with a limit of detection of ≥ 200 parasites/µL. Once testing was done, positive RDT cassettes were stored at ambient temperature in a sealed pouch, until further use. Since the exposure to malaria varies between age groups, the recruited individuals were age-stratified in: (i) children < 5 years; (ii) children between 5 and 18 year; and, (iii) adults > 18 years old. The individuals were inhabitants of Lambaréné (semi-urban), Fougamou (semi-urban) and surrounding rural areas (radius approximately 10 km). Of the 1008 archived RDT cassettes, 669 RDT cassettes that had both demographic data and positive readable test lines (with *P. falciparum*) were used for further investigation.

A blood sample (with parasitaemia of *P. falciparum* of 6,840,000 p/mL) obtained from a malaria patient was tenfold serially diluted with malaria-free group O + blood (lowest parasitaemia 68 p/mL). Five milliliter of each dilution (6 dilutions) was spotted on new RDTs (SD BIOLINE Malaria Ag P.f/Pan) and 10 µL was spotted on each circle of filter papers (Whatman™ 903 Protein Saver Card). All 6 RDTs and filter paper (6 circles) were kept at ambient temperature overnight before DNA extraction.

### DNA extraction

From RDTs Under sterile conditions, individual RDT cassettes were opened using scissors and forceps and the nitrocellulose strip was removed from the plastic case. After removal of the nitrocellulose strip, any plastic covering on the strip was stripped off. The proximal third of the test strip containing DNA was dissected and was subsequently used for DNA extraction as described elsewhere [29]. To avoid contamination, scissors and forceps were washed first with alcohol followed by DNA AWAY™ solution (Molecular BioProducts, San Diego, USA) each time and dried before opening a new RDT cassette. DNA extraction was done using QIAamp DNA Blood Mini Kit (Qiagen, Hilden, Germany) following manufacturer’s instruction for filter paper with elution volume of 50 µL. A positive control from laboratory-cultured parasites (*Pf*NF54) and a negative control devoid of parasite DNA was extracted from the RDT cassette.

From filter paper Half of each circle of filter paper containing approximately 5 µL of sample was cut and used for DNA extraction. The DNA extraction was done using QIAamp DNA Blood Mini Kit (Qiagen, Hilden, Germany) following manufacturer’s instruction for filter paper, elution volume was 50 µL.

### *Plasmodium* detection and quantification

Taqman probe-based Pan-*Plasmodium* Real-time PCR targeting a highly conserved region of 18S rRNA was used to detect and quantify *Plasmodium* parasites [30] without using reverse transcriptase (RT). The utilized primer and probes for the assay are described in Additional file 1: Table S1. The assays were performed using TaqMan™ RNA-to-CT™ 1-Step Kit (Thermo Fisher Scientific, Foster City, CA, USA) following manufacturer’s instructions for non-RT PCRs in a LightCycler 480 Instrument II (Roche, Basel, Switzerland). A negative control devoid of parasite DNA and a *P. falciparum* NF54 positive control were integrated in each run. The results were analysed by LightCycler^®^ 480 SW v1.5.1 software. The success of an amplification is defined by respective Cq values that are smaller or equal to 40.

### *pfcrt* genotyping by Taqman probe-based real-time PCR

The *pfcrt* genotyping for codons 72–76 was performed using previously described primers and probes [31] and details were provided in Additional file 1: Table S1. To enhance the sensitivity, the *pfcrt* locus was pre-amplified by conventional PCR and the enriched amplicon was used as template for real-time PCR to detect *pfcrt* haplotypes. In brief: for conventional PCR, a total reaction volume of 20 µL was constituted by adding 2.5 µL of template to 17.5 µL of reaction mix containing: 1 × PCR buffer, 0.4 µM of each primer, 0.25 mM dNTPs, 1U of Taq polymerase (Qiagen, Hilden, Germany). The thermal conditions were: 94 °C for 5 min followed by 25 cycles of 94 °C for 30 s, 55 °C for 30 s and 72 °C for 30 s, a final annealing at 72 °C for 10 min. This PCR was done using MyCycler (BioRad, Germany). The amplicons from the conventional PCR were used as template for the multiplex real-time PCR using three haplotype specific probes namely CQ-sensitive (CVMNK) and two CQ-resistant genotypes (CVIET and SVMNT) Additional file 1: Table S1. Multiplex PCR was performed on a LightCycler 480 Instrument II using SensiMix™ II Probe Kit (Bioline GmbH., Germany) following manufacturer’s instructions. Each sample was tested in duplicates. DNA extracted from *P. falciparum* NF54, Dd2 and 7G8 strains were used as positive controls for genotyping CVMNK, CVVIET and SVNMT haplotypes, respectively. Non-template controls as well as positive controls were included in every PCR batch. Amplification was considered as a success or positive when Cq values were equal to or smaller than 40.

**Table 1:** Prevalence of *pfcrt* haplotypes, age distribution and *msp1* genotyping result across regions

	FGM	LA	RR	Total
N (%)	122 (18.2)	125 (18.7)	422 (61.3)	669
Age				
≤ 5 years (%)	23 (18.8)	24 (19.2)	138 (32.7)	185 (27.7)
≥ 5 to 18 years (%)	80 (65.6)	58 (46.4)	208 (49.3)	346 (51.7)
Adults (> 18 years) (%)	19 (15.6)	43 (34.4)	76 (18.0)	138 (20.6)
*pfcrt*-PCR positivity (%)	109 (18.6)	108 (18.5)	368 (62.9)	585
CQ-sensitive (CVMNK) only (%)	21 (19.3)	37 (34.3)	68 (18.5)	126 (21.6)
CQ-resistance 1 (CVIET) only (%)	43 (39.4)	50 (46.3)	174 (47.3)	267 (45.6)
CQ-resistance 2 (SVMNT) (%)	0	0	0	0
Mix (R and S) (%)	45 (41.3)	21 (19.4)	126 (34.2)	192 (32.8)
*msp1* PCR positivity (%)	111 (18.7)	107 (18.1)	374 (63.2)	592
Monoclonal infection	24 (21.6)	43 (40.2)	96 (25.7)	163 (27.5)
Polyclonal infection	87 (78.4)	64 (59.8)	278 (74.3)	429 (72.5)
K1 only	18	40	88	146
MAD20 only	10	15	31	56
RO33 only	11	14	39	64
K1 + MAD20	14	10	61	85
K1 + RO33	24	17	66	107
MAD20 + RO33	8	4	14	26
K1 + MAD20 + RO33	26	7	75	108

Proportions of monoclonal and polyclonal infection were calculated within regions. Proportion of monoclonal infection in LA was significantly lower than that in the other two regions (df = 2, p-value = 0.004). Overall prevalence of CQ resistance (by summing up the prevalence of resistance and mix infection) was 65.7% in LA and 81.3% in rural regions (including FGM)

R, chloroquine resistance; S, chloroquine sensitive; FGM, Fougamou; LA, Lambaréné; RR, rural regions; CQ, chloroquine

### Genotyping of *msp1* locus

Amplification of *msp1* gene using conventional PCR The *msp1* gene was chosen as a target for genotyping in this study. A nested PCR was performed using published pairs of primers [32]. Primary PCR amplified the conserved region in *msp1* followed by a nested PCR which amplified the family specific block 2 of the *msp1* gene: K1, MAD20 and RO33 (sequences of primers are listed in Additional file 1: Table S1). Primary PCR was set with total volume of reaction of 20 µL containing 5 µL of template mixed with 15 µL of master mix (1× AmpliTaq Gold buffer, 1.5 mM MgCl_2_, 0.25 mM dNTPs, 1 U of Ampli Taq Polymerase and 250 nM each primer). The thermal cycling reaction was performed using a MyCycler (BioRad, Germany) for 10 min at 94 °C followed by 35 cycles of 94 °C in 15 s, 58 °C for 30 s, 72 °C for 1 min and 72 °C for 10 min for the final extension. The PCR product of the primary PCR was used as template for nested PCR. For nested PCR, three sets of reaction mixes were made with the total volume of reaction of 20 µL for three pairs of primers. The reaction mixes contain 2.5 µL of template, other components were added with the same concentration as used in the primary PCR. Thermal conditions of the nested PCRs were also similar to that of the primary PCR except the annealing temperature was at 61 °C. DNA of *P. falciparum* NF54, Dd2, and 7G8 strains were used as positive controls for three *msp1* families K1, MAD20, and RO33, respectively.

Amplicon sizing using capillary electrophoresis Amplicons were sized using an automated capillary gel electrophoresis QIAxcel Advance system (QIAGEN, Hilden, Germany) according to manufacturer’s instructions. In brief, the QIAxcel DNA High Resolution Kits (cat. no. 929002) were used. Samples were run on 96-well plates, with OM400 protocol using QX DNA Size Marker 50–800 bp (50 µL) v2.0 (Qiagen, cat. no. 929561) and QX Alignment Marker 15 bp/1 kb (Qiagen, cat. no. 929521). Interpretation was done with software QIAxcel ScreenGel v1.5.0. PCR product plates with positive controls that were positive with a single peak (NF54: 241 ± 3 bp, Dd2: 205 ± 3 bp and 7G8: 153 ± 3 bp) were included in the analysis. Each peak represents an allele and was excluded from the analysis if smaller than 100 bp or contributes less than 10% of total peak height. Within one sample, peaks with less than 6 bp difference were interpreted as one allele and average size was used for further analyses.

### Statistical analysis

All statistical analyses were computed with R software (version 3.5.1). Where applicable, 95% confidence intervals are given. Parasitaemia was calculated by extrapolating cycle threshold (Ct) values and standard curves based on linear regression analysis (assuming the amount of blood spotted on each RDT and calibrators was identical: 5 µL). All positive PCRs with Ct values > 35 and < 40 were considered negative (0 parasite/mL) using a regression model and excluded from analysis. Paired sample t-test was used to compare Ct-values of PCRs run on RDT and filter paper serial dilutions.

The MOI was defined as the mean number of *P*. *falciparum* genotypes per infected individual. The MOI was calculated as the proportion of the total number of *P*. *falciparum msp1* genotypes and the total number of PCR positive isolates. Isolates with more than one genotype were considered as a polyclonal infection while the presence of a single allele was considered as a monoclonal infection.

Mean number of alleles detected was displayed, but comparisons of MOI between age or location groups were done using the non-parametric Kruskal–Wallis H and Wilcoxon rank-sum tests. These tests were also used to compare log_10_ transformed parasitaemia density between age groups. Logistic regression models were used in this study: to check whether there was an effect of age and location on MOI (Poisson regression model), on *pfcrt* genotype (Binomial regression model) and to analyse the relationship between time of sample storage and positivity of qPCR (Binomial regression model). A linear regression model was used to assess the relationship between age and parasite density. A two-sided p-value of < 0.05 was considered statistically significant.

## Results

### Demographic details of studied population

Of the 669 individuals from whom the positive RDTs were collected, 332 (50%) were female and the median age was 8 years (interquartile range (IQR): 4–15). There were 185 (28%) children younger than 5 years, 346 (52%) aged between 5 and 18 years and 138 (21%) aged > 18 years. A total of 125 (18.7%) samples were from Lambaréné, 122 (18%) from Fougamou and 422 (63%) from rural areas as shown in Table 1. The mean time from sample collection to extraction was 113.2 days (range 9–231 days).

### Performance of RDTs as a source of DNA for quantitative PCR

**Fig. 1:**
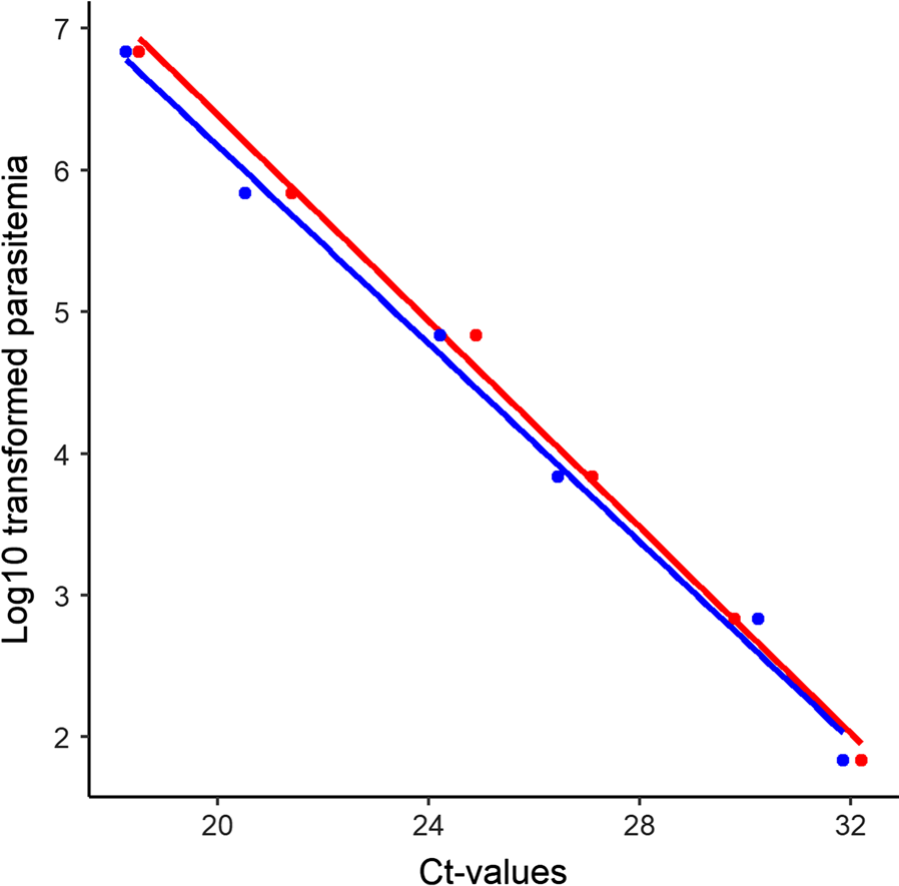
Comparison of Ct-values for different parasitaemia between two groups of samples RDT (red) and filter paper (blue). The PCRs were done in triplicate. The dots represent the average Ct-values of triplications. The smooth lines visualized the linear models between Ct-values and log_10_ transformed parasitaemia of RDT group (red, adjusted R^2^: 0.9944) and filter paper group (blue, adjusted R^2^: 0.9892)

Two series of diluted samples including 6 RDTs and 6 circles of filter paper were run on 18S real-time PCR in triplicates in order to compare the performance of these DNA sources. PCRs were positive with all samples including lowest parasitaemia samples (70 parasite/mL). The PCR results showed no differences in Ct-values between two groups of samples: RDT and filter paper (df = 5, p-value = 0.1) (Fig. 1).

**Fig. 2:**
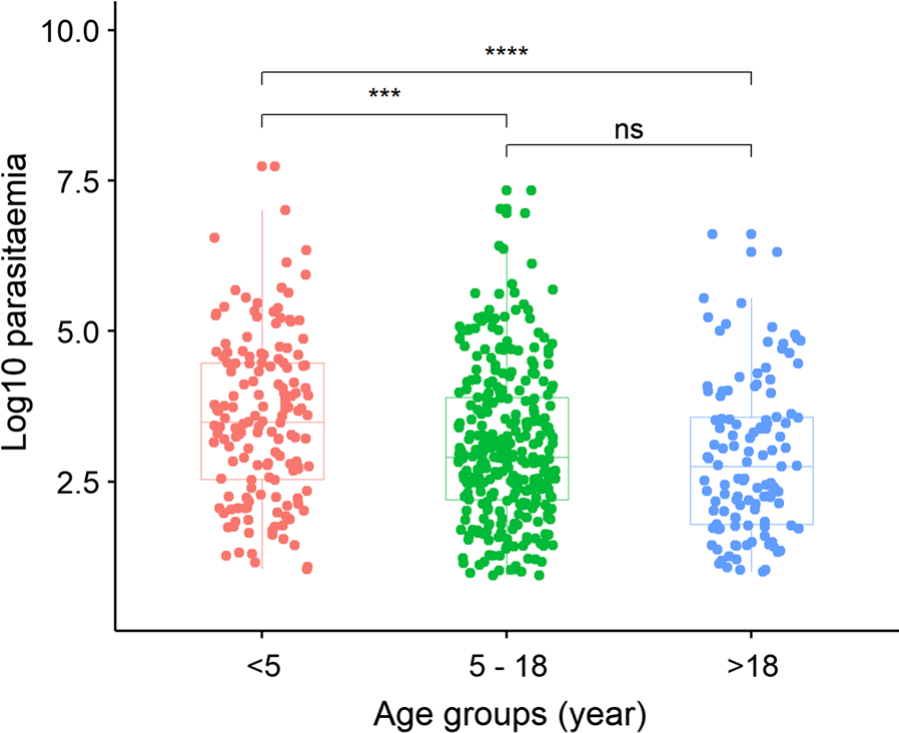
Parasitaemia across age groups. Y axis represents the log 10 transformed parasitaemia. The significance levels were shown as: significant (***: < 0.001, ****: < 0.0001) and non-significant (ns)

Median log_10_ parasitaemia in the age group < 5 years was 3.50 (IQR: 2.5–4.5), in the other two age groups 5 to 18 years and > 18 years, were 2.91 (IQR: 2.2–3.9) and 2.75 (IQR: 1.8–3.6), respectively (Fig. 2) and parasitaemia in the first group was significantly higher than the two latter ones. PCR amplification success rates were 97, 88.5 and 87% for 18S, *msp1* and *pfcrt*, respectively (Table 2). Longer time of sample storage did not lower the performance of RDTs as a source of DNA for 18 s PCR (p-value = 0.78) (Table 2).

**Table 2:** Proportion of positivity of molecular assays using DNA extracted from RDTs

	Time of sample storage				
	< 1 month (N = 138)	1–3 months (N = 57)	3–6 months (N = 361)	> 6 months (N = 113)	Total (N = 669)
Assay					
18 s PCR	135 (97.8%)	52 (91.2%)	353 (97.8%)	106 (93.8%)	646 (96.6%)
*pfcrt* PCR	120 (87%)	49 (86%)	314 (87%)	102 (90.3%)	585 (87.4%)
*msp1* PCR	112 (81.2%)	52 (91.2%)	322 (89.2%)	106 (93.8%)	592 (88.5%)

PCR, polymerase chain reaction; *pfcrt*, *Plasmodium falciparum* chloroquine resistance transporter; msp, merozoite surface protein; N, sample size

### *msp1* genotyping reveal high population diversity of *Plasmodium falciparum*

Out of 592 isolates positive for *msp1*, the K1, MAD20 and RO33 alleles were observed at the following proportions K1: 75% (n = 446), MAD20: 47% (n = 275) and RO33 51% (n = 305). The highest number of alleles detected in a single infection was 11. The MOI of the studied population was: 2.6 (95% CI 2.5–2.8).

**Fig. 3:**
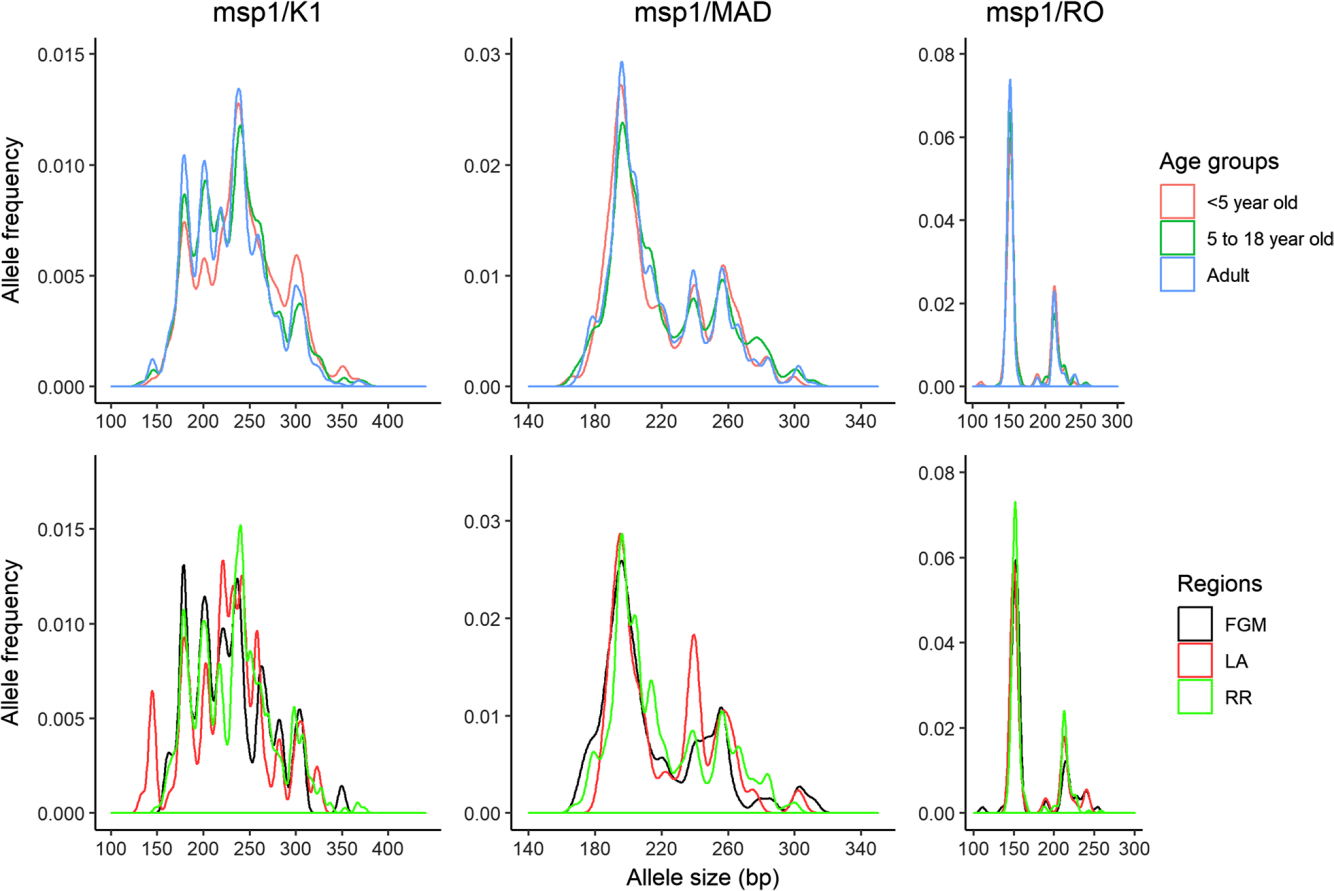
Frequency of alleles across age groups and region. The upper lane: frequency of *msp1* alleles across age groups. The lower lane: *msp1* alleles frequency across 3 studied areas. *RR* rural areas, *FGM* Fougamou, *LA* Lambaréné

Based on *msp1* genotyping, 27.5% (n = 163) were monoclonal infections Table 1. The major proportion of infections were with *msp1*/K1 family, whereas polyclonal infections were most frequently with *msp1*/K1 + RO33 and K1 + Ro33 + MAD20, Table 1. Allele sizes of *msp1* were 133–374 bp (K1), 133–311 bp (MAD20) and 111–258 bp (RO33) (Fig. 3). There was no distinct allelic pattern for a specific age group. There were 8 samples from Lambaréné carrying K1 alleles (125–155 bp), which were not observed in other regions. The mean log parasitaemia of the 8 samples from Lambaréné was 4.5 (95% CI 3.8–5.2), which is higher than the mean log parasitaemia of the population.

**Fig. 4:**
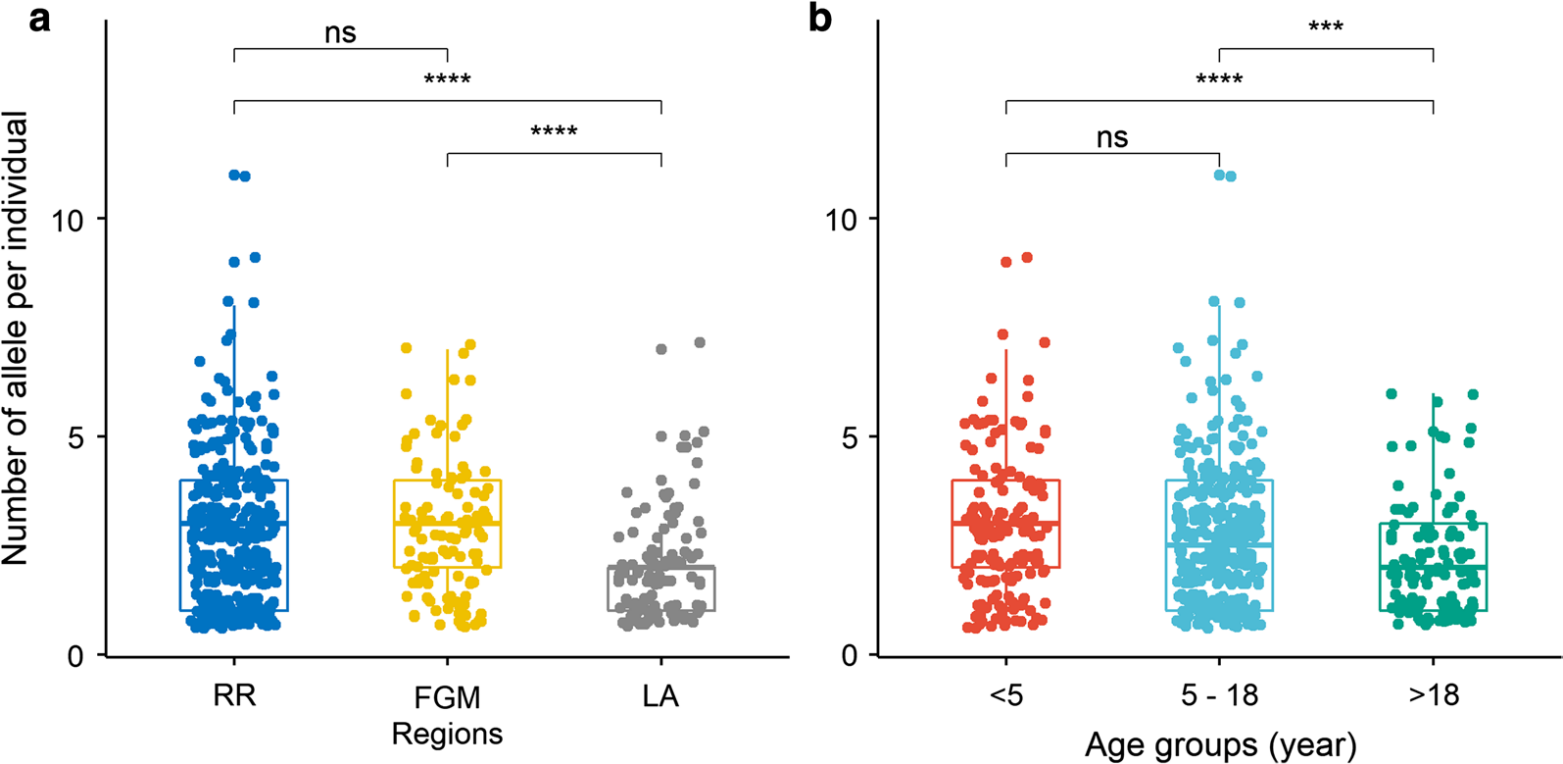
MOI across studied regions (**a**) and age groups (**b**). MOI was shown as number of allele per individual. The box plots showed the medians and inter quartile ranges (IQR). Number of allele (countable number) is visualized by jitter point. The significance levels were shown as: significant (***: < 0.001, ****: < 0.0001) and non-significant (ns). *RR* rural areas, *FGM* Fougamou, *LA* Lambaréné

The MOI for ages < 5 years was 2.8 (95% CI 2.6–3.1); for ages 5 to 18 years 2.7 (95% CI 2.5–2.9) and for ages > 18 years 2.1 (95% CI 1.9–2.4). The MOI for Lambaréné was 2.0 (95% CI 1.8–2.3), Fougamou was 2.8 (95% CI 2.5–3.1) and rural areas was 2.8 (95% CI 2.6–2.9) (Fig. 4). Both age and location were observed to associate with MOI as computed in the Poisson regression model (age: β = − 0.0046, p-values = 0.019 and location: β = − 0.314, p = 0.0004).

### Prevalence of CQ-resistant haplotypes

**Fig. 5:**
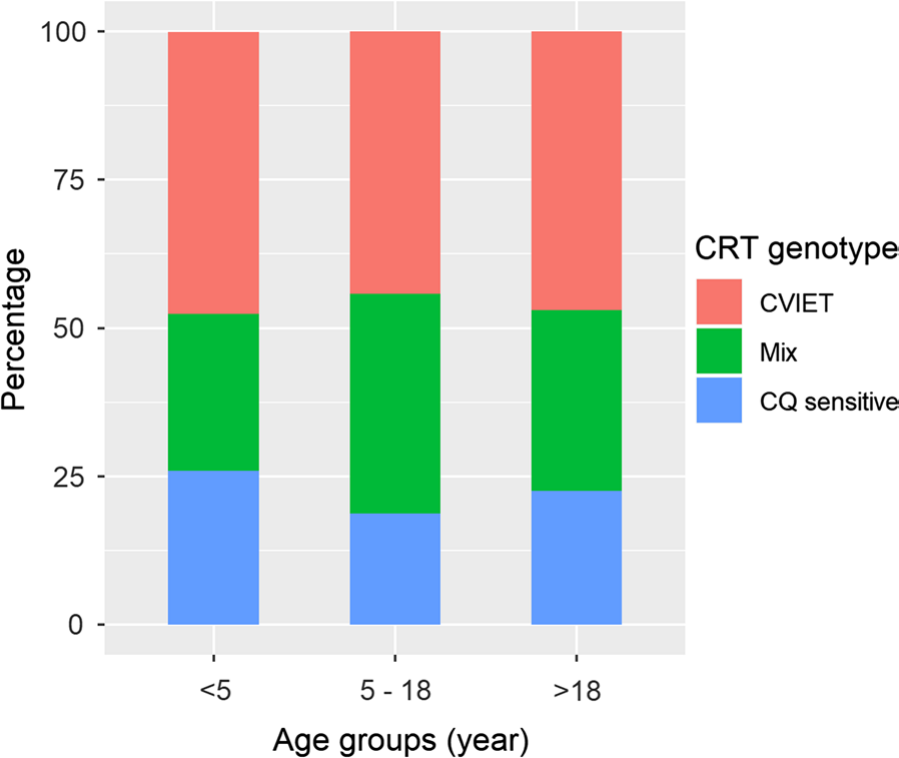
Prevalence of *Pfcrt* haplotypes across age groups. Mix infections contain both sensitive and resistant genotypes. Haplotype SVMNT was not detected in this population

In the group aged < 5 years, 26% of the samples were CQ-sensitive, 48% were resistant (CVIET genotype) and 27% were mixed infections carrying both sensitive and resistant strains (Table 1). The prevalence of sensitive, CVIET and mixed strains in other groups was 18.8, 44.2 and 37%, respectively for the age group from 5 to 18 years; 22.6, 47.0 and 30.4%, respectively for adults (Fig. 5). The overall prevalence of CQ-resistant strains was 78.5%. There was no statistically significant difference in the prevalence of CQ-resistant strains between age groups (p-value = 0.2). A statistically significant difference was observed in the prevalence of CQ-resistant genotypes between Lambaréné and the other areas (Fougamou and rural areas), 65.7 *vs* 81.3% (p-value = 0.0017) and there was no difference observed between Fougamou and rural areas Table 1. This result was further confirmed by logistic regression, in which living in Lambaréné was significantly associated with resistance of *P. falciparum* (β = − 0.809, p-value = 0.011 after correction for age).

## Discussion

This study retrospectively investigated the utility of archived RDTs with a large sample size for molecular characterization of *P. falciparum* population diversity, MOI and the distribution of CQ-resistant haplotypes. The main findings of this study are a high amplification success rate of PCRs using DNA extracted from archived RDTs which were not initially stored for this purpose, diversity of *P. falciparum* parasites and the persistence of CQ-resistant strains.

Results from pan-*Plasmodium* PCR showed that archived RDTs are a reliable source of DNA for real-time PCR, comparable to filter paper [13]. PCR using DNA extracted from both sources could detect *Plasmodium* parasites at a low density. Even approximate quantification of malaria parasites from RDT samples is possible, which adds important information beyond the RDT result. However, quantification can be biased by the unequal blood volume spotted on RDT strips during RDT preparation [33]. The log transformed parasitaemia result in this work, and therefore was used only in comparison analyses with the assumption that blood volumes were similar (on field samples and controls). The storage duration of archived samples (up to 6 months) did not compromise either with DNA quality or the amplification success rate. Age of the host and the intensity of exposure to the parasites are the major factors which influence the risk of developing parasitaemia and disease by inducing and regulating the host immunity to malaria, therefore higher parasitaemia in children than in adults has been observed [34].

There have been many approaches used to estimate the diversity of malaria parasite populations; those based on PCR and targeting the length polymorphic genes have been commonly used. The performance of these approaches relies mainly on the resolution of the sizing system and could be enhanced by using capillary electrophoresis. This study is the first one performed in Lambaréné using the high resolution, automated capillary gel electrophoresis by QIAxcel Advanced System to investigate the genetic diversity of *P. falciparum msp1* gene in field isolates. This approach provides a highly discriminatory power since the two fragments can be distinguished by three base-pairs difference, and is able to detect up to 11 alleles in an infection.

MSP1 of *P. falciparum* is a major protein, which plays an important role in the invasion of the parasite into the host’s red blood cell. Genotyping malaria parasites based on merozoite surface genes may contribute to the understanding of selection pressure. This work shows the differences in multiplicity of malaria infections between age groups and geographic regions and a high level of polymorphisms of *msp1* in the studied population. The overall MOI in the studied population was 2.6 (2.0 for Lambaréné and 2.8 for Fougamou and rural areas), which is similar to previous studies conducted in Gabon [35] and lower compared to 2000 [36], before the application of ACT as the first-line therapy. Innate immunity against malaria developed after exposure to different *P. falciparum* infections, which directly related to age and transmission intensity, therefore, allelic diversity can decrease with increasing age of the infected individual. After being corrected for the effect of age, difference in geographic location has an effect on MOI. One reason could be that less diversity of parasites within the mosquito or different drug pressures might cause disappearance and re-appearance of parasite clones in patients [36].

Product of the *msp1/K1* and *msp1/MAD20* gene families have been identified as candidates for *P. falciparum* vaccines [37]. In this studied population, K1 was predominantly detected followed by RO33 and MAD20 families. RO33 appeared to be not as polymorphic as K1 and MAD20 families and there were no regional or age specific alleles. This finding is consistent with results from other studies performed in different settings and geographic regions [36, 38]. The predominant allele size of K1 *P. falciparum* was around 240 bp (26.7%), similar to the length of the *msp1* gene of strain NF54 (241 bp), which is being used as a vaccine candidate in some clinical trials in Lambaréné.

Gabon is a country where prevalence of biomarkers of CQ resistance remained high although a partial reduction was noticed in the last decade [39]. In Moyen-Ogooué, CQ-resistant haplotypes were significantly less common in Lambaréné and surrounding areas (radius approximately 10 km) than in Fougamou and rural areas. Prevalence of CQ-resistant haplotypes was age independent. All of the CQ-resistant haplotypes were CVIET (triple mutation at codons 74, 75 and 76), no sample carried haplotype SVMNT (double mutation at codons 72 and 76). This result highlights the need for further analyses to better understand the mechanism behind the maintained high prevalence of CQ resistance. Amodiaquine has been linked to the persistence of CQ-resistant strains since its use in combination with artesunate may have resulted in continued selection of mutant *pfcrt* haplotypes [39].

One limitation of this work is that it did not include negative field RDTs, therefore infections with low parasite density, which were negative by RDT could not be analysed. That limitation could hinder documenting the existence of circulating parasite genotypes which might also contribute towards transmission intensity and drug resistance.

## Conclusions

This study showed that RDTs are a reliable source of DNA for *P. falciparum* detection and genotyping analyses using PCR and capillary electrophoresis. The parasite population in the studied regions is highly diverse and prevalence of *pfcrt* in Gabon remains high. Prevalence of molecular markers of CQ resistance is age independent, but regionally dependent, which may indicate the effect of transmission intensity on the prevalence of resistance rather than acquired immunity. The work also suggests the possibility of quantifying *Plasmodium* parasites in RDTs, especially when the volume of blood used is uniform.

## Supplementary information

**Additional file 1: Table S1.** Primer and probe sequences.

## Data Availability

The datasets used during the current study are available from the corresponding author on reasonable request.

## Abbreviations

ACT: artemisinin-based combination therapy
CERMEL: Centre de Recherches Médicales de Lambaréné
CQ: chloroquine
Ct: cycle threshold
DNA: deoxyribonucleic acid
dNTPs: deoxynucleotide Triphosphates
EDTA: ethylenediaminetetraacetic acid
FGM: fougamou
LA: lambaréné
MOI: multiplicity of infection
*msp1*: merozoite surface protein 1
NAT: nucleic acid test
PCR: polymerase chain reaction
pfcrt: *Plasmodium falciparum* chloroquine resistance transporter
RDT: rapid diagnostic test
RR: rural region
RT: reverse transcriptase
WHO: World Health Organization
