# Supplementary material for: DNA recovery from archived RDTs for genetic characterization of Plasmodium falciparum in a routine setting in Lambaréné, Gabon
Source: Malar J. 2019 Oct 2;18:336. doi: 10.1186/s12936-019-2972-y (PMC6775649; doi:10.1186/s12936-019-2972-y)
Supplement: Supplementary file 1 — Additional file 1: Table S1. Primer and probe sequences. [file 12936_2019_2972_MOESM1_ESM.docx]

**Additional file 1: Table S1.** **Primer and probe sequences**

| *msp1* | Primary PCR | | A | AAGCTTTAGAAGATGCAGTATTGAC |
| --- | --- | --- | --- | --- |
|  |  |  | B | ATTCATTAATTTCTTCATATCCATC |
|  | Nested PCR | K1 | Fwd | AAATGAAGAAGAAATTACTACAAAAGGTGC |
|  |  |  | Rev | GCTTGCATCAGCTGGAGGGCTTGCACCAGA |
|  |  | MAD20 | Fwd | AAATGAAGGAACAAGTGGAACAGCTGTTAC |
|  |  |  | Rev | ATCTGAAGGATTTGTACGTCTTGAATTACC |
|  |  | RO33 | Fwd | TAAAGGATGGAGCAAATACTCAAGTTGTTG |
|  |  |  | Rev | CATCTGAAGGATTTGCAGCACCTGGAGATC |
| *pfcrt* | Pre-amplification | | Fwd | TGGTAAATGTGCTCATGTGTTT |
|  |  |  | Rev | AGTTTCGGATGTTACAAAACTATAGT |
|  | RT-PCR | | Fwd | TGGCTCACGTTTAGGTGGAGGTTCTTG |
|  |  |  | Rev | ACTGAACAGGCATCTAACATGGATATAGC |
|  | Probes | | CVMNK | TGTGTAATGAATAAAATTTTTGCTAA |
|  |  |  | CVIET | TGTGTAAT**TG**A**A**A**C**AATTTTTGCTAA |
|  |  |  | SVMNT | **A**GTGTAATGAATA**C**AATTTTTGCTAA |
| 18S PCR | PLU3 Forward | | | GCTCTTTCTTGATTTCTTGGATG |
|  | PLU3 Reverse | | | AGCAGGTTAAGATCTCGTTCG |
|  | PLU3 Probe | | | VIC-ATGGCCGTTTTTAGTTCGTG-NFQ-MGB |

Red letters represent the mutation points

Fwd: forward primer

Rev: reverse primer
